# Sources of Phoneme Errors in Repetition: Perseverative, Neologistic, and Lesion Patterns in Jargon Aphasia

**DOI:** 10.3389/fnhum.2017.00225

**Published:** 2017-05-04

**Authors:** Emma Pilkington, James Keidel, Luke T. Kendrick, James D. Saddy, Karen Sage, Holly Robson

**Affiliations:** ^1^School of Psychology and Clinical Language Sciences, University of ReadingReading, UK; ^2^School of Psychology, University of SussexBrighton, UK; ^3^Department of Allied Health Professions, Sheffield Hallam UniversitySheffield, UK; ^4^Centre for Health and Social Care, Sheffield Hallam UniversitySheffield, UK

**Keywords:** repetition, aphasia, neologisms, perseveration, voxel-lesion symptom mapping, Jargon aphasia

## Abstract

This study examined patterns of neologistic and perseverative errors during word repetition in fluent Jargon aphasia. The principal hypotheses accounting for Jargon production indicate that poor activation of a target stimulus leads to weakly activated target phoneme segments, which are outcompeted at the phonological encoding level. Voxel-lesion symptom mapping studies of word repetition errors suggest a breakdown in the translation from auditory-phonological analysis to motor activation. Behavioral analyses of repetition data were used to analyse the target relatedness (Phonological Overlap Index: POI) of neologistic errors and patterns of perseveration in 25 individuals with Jargon aphasia. Lesion-symptom analyses explored the relationship between neurological damage and jargon repetition in a group of 38 aphasia participants. Behavioral results showed that neologisms produced by 23 jargon individuals contained greater degrees of target lexico-phonological information than predicted by chance and that neologistic and perseverative production were closely associated. A significant relationship between jargon production and lesions to temporoparietal regions was identified. Region of interest regression analyses suggested that damage to the posterior superior temporal gyrus and superior temporal sulcus in combination was best predictive of a Jargon aphasia profile. Taken together, these results suggest that poor phonological encoding, secondary to impairment in sensory-motor integration, alongside impairments in self-monitoring result in jargon repetition. Insights for clinical management and future directions are discussed.

## Introduction

Neologistic Jargon aphasia is an acquired language disorder characterized by severely distorted speech output. Production in Jargon aphasia is fluent but underspecified and contains numerous non-word errors, rendering it hard to comprehend. Prognosis in Jargon aphasia is poor, with reports of declining vocabulary size and mixed therapeutic outcomes (e.g., Panzeri et al., 1987; Robson et al., 1998a,b; Eaton et al., 2011; Bose, 2013). Perseveration, repeated patterns of phonological distortion, frequently co-occurs with Jargon aphasia and is particularly evident during elicitation tasks such as serial repetition.

Neurobiologically, the repetition of a word requires the transformation of sensory information into motor activation. Traditional neurological accounts of impaired repetition posit damage to the white matter tracts—particularly the arcuate fasciculus—connecting posterior and anterior language areas as the source of breakdown (Geschwind, 1965). Recent neuroimaging and stimulation work has expanded this dorsal network to include cortical regions; namely the inferior supramarginal gyrus (SMG) and posterior superior temporal gyrus (pSTG; Anderson et al., 1999; Quigg and Fountain, 1999) including area Spt at the boundary of the inferior parietal and superior temporal lobes, which includes portions of the planum temporale (Hickok et al., 2003, 2009; Hickok and Poeppel, 2004). In repetition, the pSTG plays a perceptual role analyzing phonetic and phonemic information in the speech stream (Buchsbaum et al., 2001; McGettigan et al., 2010; Deschamps and Tremblay, 2014). This phonological information is transformed into motor responses for articulatory processes, a function proposed to be supported by area Spt (Hickok and Poeppel, 2004; Warren et al., 2005; Hickok, 2009; Buchsbaum et al., 2011; Hickok et al., 2011). Area Spt has direct structural connectivity with motor and frontal regions, including the pars opercularis and premotor cortex which are associated with the articulatory components of speech production (Isenberg et al., 2012; Basilakos et al., 2015; Itabashi et al., 2016). The SMG is also proposed to support encoding for production (Ravizza et al., 2004; Trébuchon et al., 2013; Mesgarani et al., 2014) but is more prominently associated with auditory short-term memory/working memory functions (Paulesu et al., 1993; Henson et al., 2000) which support the temporary maintenance of phonological information during the repetition process.

Convergent with the neurobiological account, cognitive neuropsychological and psycholinguistic models highlight a phonological pathway for repetition. In addition, many models allow a further repetition route via a semantic pathway (McCarthy and Warrington, 1984; Hillis and Caramazza, 1991; Hanley and Kay, 1997; Hanley et al., 2004; Dell et al., 2007; Nozari et al., 2010). Word repetition is commonly impaired in aphasia, and has classically been used as a diagnostic screening test (Kaplan, 1983). However, repetition errors do not occur in all aphasic conditions. For example, individuals with isolated semantic impairment such as those with transcortical sensory aphasia or semantic dementia have preserved repetition abilities (Boatman et al., 2000; Jefferies and Lambon Ralph, 2006; Hodges et al., 2008). Where repetition errors do occur, they tend to be phonological in nature, with a comparative scarcity of purely semantic errors (Martin et al., 1994; Martin, 1996; Hanley et al., 2002). These behavioral patterns are consistent with a neurobiological mechanism predominantly engaging sensory-motor integration functions with relatively less weight on semantic processes (Moritz-Gasser and Duffau, 2013). Non-words are one form of phonological repetition error which are particularly frequent in individuals with Jargon aphasia. Non-words can range from mild phonemic substitutions of acoustically or articulatory similar phonemes (e.g., village—/vilti:/), typically referred to as phonological paraphasias, to severe distortions which bear little resemblance to target phonology (e.g., rocket—/waɪæpəl/), typically referred to as neologisms. Perseverative errors, the repeated intrusion of phoneme strings or syllabic patterns, have been noted to occur alongside neologistic production in Jargon aphasia (Buckingham et al., 1978; Moses et al., 2004). A fourth type of error commonly observed is referred to as a formal error, which occurs when an alteration in the phonological structure of a word results in a real word error (e.g., cot—/kəʊt/). There has been considerable research into the underlying causes of non-word and perseverative errors in repetition and other production modalities. Much evidence points to a single impairment source for paraphasias, neologisms and perseverative errors, with different error types reflecting a range of severity (Dell et al., 1997; Schwartz et al., 2004; Martin and Dell, 2007; Olson et al., 2007, 2015; Buckingham and Buckingham, 2011). The predominant hypothesis indicates a disruption in lexical and phonological processes, during which weak and aberrantly spreading activation can result in non-target phonology being selected for production. Non-word production is modulated by word length and word frequency, suggestive of a single lexico-phonological source, generating errors with a range of severity (Olson et al., 2007, 2015; Nozari et al., 2010). Non-word accuracy range adheres to a normal distribution, thereby suggesting that a single underlying source generates errors of varying severity (Olson et al., 2007). An alternative hypothesis is that paraphasic and neologistic non-words are independent error types whereby neologisms are produced when lexical retrieval fails and a random or idiosyncratic phoneme string is generated for output (Butterworth, 1979; Buckingham, 1990; Moses et al., 2004; Eaton et al., 2010). Such production would give rise to two separate error populations; one with very limited target relatedness and the other with high target overlap, thereby conforming to a bimodal distribution.

The source of perseveration errors is also controversial. The predominant hypothesis states that weak target activation or phonological encoding allows recently used and, therefore, the most active representations, to override the current target (Hirsh, 1998; Ackerman and Ellis, 2007; Moses et al., 2007a; Eaton et al., 2010; Buckingham and Buckingham, 2011). As such, perseverative, paraphasic and neologistic errors are hypothesized to have a common source. The co-occurrence of perseverative and non-perseverative non-word errors supports this hypothesis (Martin and Dell, 2007; Moses et al., 2007b). An alternative hypothesis posits that errors arise from disruption of inhibitory processes and a failure of post-activation suppression (Yamadori, 1981; Sandson and Albert, 1984; Santo Pietro and Rigrodsky, 1986; Papagno and Basso, 1996; Stark, 2007). Concurrent inhibition and encoding deficits have been identified in some dysgraphic individuals indicating that these mechanisms are not mutually exclusive (Fischer-Baum and Rapp, 2012). However, it is unclear whether such inhibitory mechanisms are a specific feature of the phonological encoding system or a domain-general cognitive function and whether different mechanisms operate more strongly in different subtypes of aphasia. A significant challenge in distinguishing between non-word error and perseveration hypotheses within the neologistic Jargon aphasia population comes from the relative rarity of the condition, which has resulted in small scale case-series investigations or single case studies. This results in difficulty applying psycholinguistic patterns to the wider Jargon aphasia population.

Despite this, evidence from lesion-symptom mapping is currently consistent with the proposed impairment in phonological encoding put forward by computational modeling and neuropsychological investigations. Repetition errors in chronic aphasia have been associated with lesions affecting the left inferior parietal lobe (IPL; Fridriksson et al., 2010), the left posterior temporo-parietal cortex (Baldo et al., 2012), and area Spt (Rogalsky et al., 2015) similarly interpreted as a disruption to sensory-motor integration (including phonological encoding). However, lesion-symptom mapping, modeling and neuropsychological evidence is not currently directly comparable. Lesion-symptom mapping repetition studies currently contain few or no individuals with jargon-type repetition impairments and predominantly include those with conduction-like repetition deficits (Baldo et al., 2012; Rogalsky et al., 2015), reducing the applicability of these results to the jargon population. As such, the possibility remains that more “peripheral” aspects of the repetition system, such as perceptual auditory-phonological or articulatory processing, may contribute to jargon repetition. An impairment in perceptual analysis is consistent with the majority of individuals with Jargon aphasia also displaying Wernicke's-type aphasia associated with auditory-phonological processing impairments (Robson et al., 2012, 2013, 2014) and the association of neologistic production and impairments in self-monitoring (Kinsbourne and Warrington, 1963; Maher et al., 1994; Marshall et al., 1998). Perceptual and articulatory processes are also not captured in computational modeling which focuses on core linguistic components of semantic, lexical and phonological processing. A further possibility is that no single process or neural region results in the deficit. Rather, jargon repetition may occur following damage to multiple components of the repetition network, resulting in the severe distortions observed in the condition. Investigating the lesion profiles associated with non-word and perseverative errors in a large cohort is required to explore these hypotheses.

In the current study, we use a combination of psycholinguistic and lesion-symptom mapping analyses to explore the cognitive and neurobiological underpinnings of jargon repetition deficits. The target relatedness and distribution of non-word errors are analyzed to distinguish the default generation and phonological encoding hypotheses. Patterns of perseveration are examined and the co-occurrence of perseveration and non-perseveration errors is explored to determine whether these error types share a common source. Whole brain and region of interest lesion-symptom mapping analyses are used to explore the contribution of the wider dorsal repetition network to neologistic Jargon aphasia.

## Methods

Ethical approval for the current study was given by the Multicenter NHS Research Ethics Committee, the NHS East of England Research Ethics Committee and the University of Reading School of Psychology Research Ethics Committee.

### Participants

We report data from 46 individuals with aphasia (female *n* = 15), mean age 69.7 years (σ = 12.24; range = 31–93), mean time post onset 35 months (σ = 47.63; range = 5–204), see Table 1. Aphasia profile was assessed with the Boston Diagnostic Aphasia Examination—Short Form (Goodglass et al., 2001). Percentile scores for auditory comprehension, repetition (word and sentence) and fluency subtests are presented in Table 1. Twenty individuals presented with Wernicke's aphasia, four with conduction aphasia, four with anomic aphasia and two with transcortical sensory aphasia. In the non-fluent categories, four participants were classified as Broca's type aphasia, with one individual classified as transcortical motor aphasia. Four individuals were classified as mixed aphasic and the remaining six were unable to be classified as the necessary BDAE data were unavailable. Different individuals were entered into behavioral and neuroimaging analyses based on analysis criteria discussed below.

**Table 1:** **Demographic, imaging and BDAE information**.

					**BDAE centiles**		
**Pt code**	**Age (years)**	**Time post stroke (months)**	**Gender**	**Imaging**	**Comprehension**	**Fluency**	**Repetition**
1	55	24	M	3T	n/a	n/a	n/a
2	70	96	M	3T	n/a	n/a	n/a
3	80	8	F	Clinical CT	30	n/a	n/a
4	54	145	F	n/a	38	n/a	45
5	56	22	M	3T	77	n/a	15
6	75	132	F	3T	58	7	60
7	63	144	M	3T	48	13	40
8	31	15	M	n/a	48	20	20
9	68	108	M	3T	87	30	65
10	81	8	M	Clinical CT	15	30	25
11	59	14	M	3T	10	38	13
12	68	24	M	3T	9	42	7
13	65	108	F	3T	50	48	45
14	74	6	M	n/a	12	51	13
15	69	15	F	n/a	33	55	10
16	72	204	M	3T	100	62	60
17	73	6	M	3T	3	63	<1
18	62	84	M	3T	n/a	63	n/a
19	78	72	F	3T	5	68	10
20	53	7	M	n/a	15	68	<1
21	64	6	M	n/a	10	68	15
22	66	10	M	3T	5	70	25
23	49	24	F	3T	70	70	60
24	81	7	F	Clinical CT	18	75	15
25	85	9	F	n/a	<1	75	<1
26	86	13	M	3T	10	80	7.5
27	88	9	M	Clinical MRI	42	80	65
28	73	13	F	3T	10	83	10
29	60	5	M	3T	7	84	8
30	77	24	M	3T	40	90	25
31	71	72	M	3T	7	90	1
32	70	42	M	3T	45	100	28
33	59	6	M	3T	17	100	20
34	75	12	M	Clinical MRI	28	100	5
35	78	9	F	3T	73	100	80
36	83	9	F	Clinical CT	48	100	60
37	93	9	F	Clinical CT	67	100	80
38	68	9	M	Clinical CT	55	100	50
39	80	9	F	n/a	25	100	20
40	71	9	M	Clinical MRI	50	100	80
41	82	9	M	Clinical MRI	64	100	30
42	76	14	M	3T	13	100	1
43	74	9	M	3T	57	100	50
44	57	9	M	3T	15	100	10
45	86	13	F	Clinical CT	3	100	15
46	49	5	M	3T	67	100	60

### Neuroimaging

**Figure 1:**
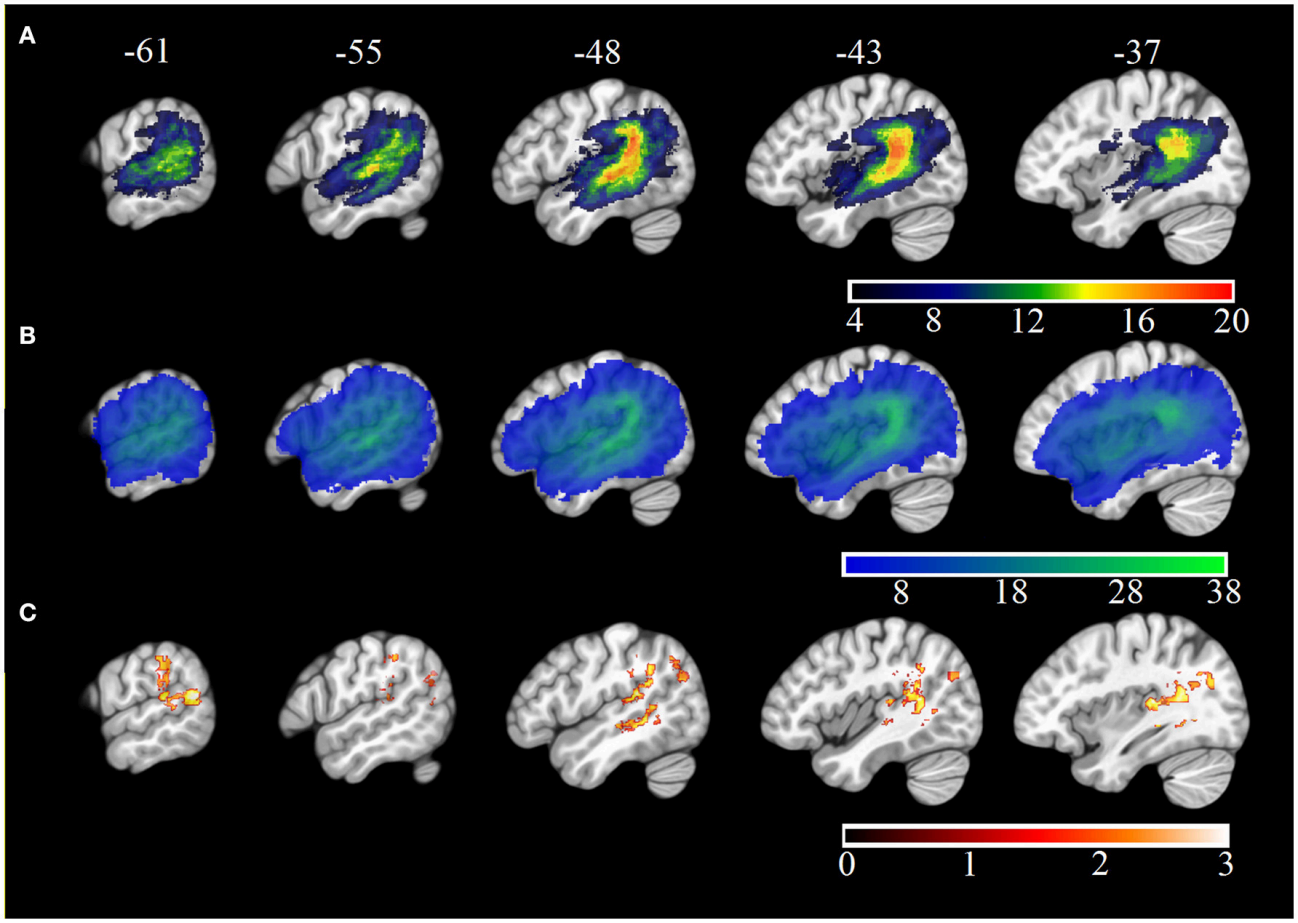
**MNI X-coordinate displayed above**. **(A)** Lesion overlap map for Jargon aphasia participants for whom imaging was available. Color bar indicates number of individuals with lesion at each voxel. **(B)** Lesion overlap map for all aphasia participants for whom imaging was available. Color bar indicates number of individuals with lesion at each voxel. **(C)** VLSM results (threshold ≤ 0.05) showing voxels significantly associated with jargon score. Color bar indicates t-statistic.

Neuroimaging data were available for 38 participants (see Table 1). 3T T1w research MRI scans were collected for 27 individuals. Scans were collected across different studies and, as a result, protocols varied. Clinical imaging scans were available for the remaining 11 participants. Only scans which were carried out after 24 h post stroke onset were included in the analysis, to avoid significant underestimation of the extent of the stroke. Lesions were manually delineated by lesion drawing in native space. The native lesion masks were used for cost-function masking during normalization. Normalization was implemented in the SPM Clinical toolbox (Rorden et al., 2012). Normalization parameters were applied to the native lesion masks which were subsequently manually checked for normalization accuracy. Lesion overlap maps for the whole aphasia group and for the Jargon aphasia subgroup are presented in Figures 1A,B. Lesions were observed throughout the entire left MCA territory in the aphasia group as a whole, with peak lesion overlap in the temporoparietal junction including the superior temporal gyrus (STG) and sulcus and SMG, in both the whole group and Jargon subgroup.

### Repetition tasks

All participants completed an 80 item word repetition task. Sixteen participants completed the word repetition test from the PALPA (Psycholinguistic Assessment of Language Processing in Aphasia, subtest 9: Kay et al., 1996) and 30 participants completed an in-house 80 item repetition test. The 80 items were administered either continuously or in shorter blocks, if a participant was perceived to require a break. The experimenter provided repetitions when requested.

### Recording and error coding

All response data were transcribed into broad phonemic transcription. When multiple responses were given per item, the final stressed response was accepted. All transcriptions were then converted into DISC symbols (1:1 phoneme: symbol correspondence, e.g., IPA = [i:], DISC = [i]); to enable automated data extraction via Microsoft excel and MATLAB. Responses were categorized following criteria used by Moses et al. (2004). Non-lexical responses were classified as non-words. Lexical errors were labeled according to their target relationship, and were classed as either formal (either identical first phoneme or fifty percent phonology overlap with target), semantic (semantically related to target), mixed (semantically and phonologically related to target word form), unrelated (real word error that did not share an obvious relationship to target), no response (individual indicated they could not provide an answer or did not respond) or circumlocution (individual provides information about the item by talking around it but not naming it).

### Analysis summary

Four different analyses were undertaken to explore behavioral patterns in jargon production. Phonological accuracy of non-words was explored, using the Phonological Overlap Index measure (POI: Schwartz et al., 2004) and non-word accuracy distributions were examined using the Kolmogorov-Smirnov test. Perseverative patterns were analyzed, using the Intrusion Perseveration Probability (IPP) measure, adapted from Cohen and Dehaene (1998) and the relationship between perseverative and non-perseverative non-words was explored, using a correlation analysis. Voxel-lesion symptom mapping (VLSM) and follow-up region-of-interest (ROI) analyses were used to investigate the relationship between jargon production and lesion profiles.

### Phonological accuracy in neologistic jargon

The degree to which neologistic errors are produced with reference to target phonology was investigated using the POI measure (Schwartz et al., 2004; Bose, 2013). The POI for each non-word repetition response was calculated using the formula:

$$\begin{array}{l} POI=\left(n\;phonemes\;shared\;between\;target\;and\;response\right)\times \;2\\ /\left(n\;phonemes\;in\;target+n\;phonemes\;in\;response\right). \end{array}$$

A value of 0 indicates no overlap with target phonology and a value of 1 indicates complete overlap between the target and response. Non-word responses were then assigned to a paraphasic (>0.51 POI) or neologism (≤0.5 POI) error category (Schwartz et al., 2004). The target relatedness of neologistic errors was compared to a chance rate derived from null distributions. In each null distribution, all non-word errors from all participants were randomly reassigned to a new target and a new POI calculated. To statistically compare individual and chance accuracy, an equal number of resampled responses coded as neologistic errors, were randomly extracted for each participant. The observed POI mean was compared against each resampled POI mean to derive a level of significance.

### Non-word accuracy distributions

The accuracy (POI) distribution of both non-word error types (paraphasias and neologisms) was examined using the one sample Kolmogorov-Smirnov (KS) test of normality, in order to examine whether distributions adhered to a normal curve and conformed to the single source hypothesis.

### Perseveration

The IPP measure, adapted from Cohen and Dehaene (1998), calculates how often a phonological error occurs in each of the previous 10 responses. To calculate it, every intruded/erroneous phoneme was identified. Then, how often each of these intruded phonemes was present (matched) in each of the previous 10 responses was measured. The probability was calculated by dividing the number of matched phonemes at each lag by the total number of intruded phonemes. The average IPP across the 10 lags was calculated so as to assign each individual with a perseveration value, representative of persistent patterns of phoneme intrusions. To account for breaks in administration, data were split into blocks of 20 responses and only responses 11–20 were analyzed in relation to the previous 10 responses. This method provided 40 trials per individual for analysis. Both correct and incorrect responses were included in the analysis. Six individuals (4, 12, 26, 41, 43, 44) were excluded from this analysis because their data could not be split into blocks of twenty.

### Chance perseveration

To interpret the prevalence of perseveration within the Jargon aphasia group, observed IPP values were compared against a chance rate. In the current study, all responses from all participants were randomly reassigned to a new target to create a null distribution and 40 trials were randomly selected to undergo IPP analysis. This process was repeated 1,000 times. The observed IPP score was compared against each resampled IPP score to derive a level of significance.

### Relationship between perseverative and non-perseverative non-words

For individuals who presented with fluent Jargon aphasia, the number of perseverative non-word errors was calculated using criteria from Martin and Dell (2007). A non-word was identified as a perseveration when a phoneme error was present in the previous response. Otherwise, the non-word was labeled as a non-perseveration. To accommodate administration breaks, the initial response in each subset was discounted. The association between perseverative and non-perseverative non-words was examined using Spearman's rank correlation.

### Voxel-lesion symptom mapping

All participants with an available clinical or 3T T1wimage were included in an exploratory VLSM analysis implemented in the vlsm2 matlab toolbox (version 2.3; Bates et al., 2003). This analysis uses a mass univariate general linear model approach to determine the relationship between the presence of lesion and behavior at each voxel while accounting for total lesion volume. The analysis was constrained to the left hemisphere gray and white matter regions. Results were obtained at thresholds of 0.05 and 0.01 and compared to those obtained from 1,000 permutations/null distributions. The VLSM analysis was extended using an ROI analysis. VLSM clusters significant at *p* < 0.05 and <200 voxels were identified and the percentage lesion overlap with each cluster was extracted in each participant. ROI data were used to identify the consistency of lesion-behavior associations and the strongest predictors of jargon repetition.

## Results

### Overall accuracy and error patterns

All but 4 individuals (participants 7; 38; 40; 46) displayed a repetition impairment ($\bar{\text{x}}$ = 35; σ = 23.24, range = 1–73; see Table 2). Individuals with anomic aphasia were the most accurate as a group ($\bar{\text{x}}$ = 54; σ = 28.62), followed by those with Broca's aphasia ($\bar{\text{x}}$ = 49.7; σ = 19.40), then conduction aphasia ($\bar{\text{x}}$ = 43.3; σ = 25.16). Those with Wernicke's aphasia were the least accurate as a group ($\bar{\text{x}}$ = 21.45; σ = 16.30). Across all participants, the predominant error types were non-words (1,288, 35%) and formal errors (304, 8%). The remaining four error categories (unrelated, semantic, circumlocution, no response) contributed just over 7% of the overall response rate. POI analysis indicated roughly equal numbers of neologistic and paraphasic errors (medians; paraphasias = 14; neologisms = 8.5; Mann Whitney U = 875.5; *p* = 0.153). Participants who presented with fluent speech and produced 5 or more neologistic errors during repetition were considered to present with neologistic Jargon aphasia; 25 participants met these criteria.

**Table 2:** **Number of each response type on single word repetition task**.

			**Nonwords**					
**Pt code**	**Test**	**Correct**	**Paraphasia**	**Neologism**	**Formal**	**Unrelated**	**No response**	**Other**
17	Palpa	1	8	71	0	0	0	1
44	DV	3	12	59	3	3	0	0
45	DV	4	20	38	8	8	1	2
20	Palpa	6	18	44	4	6	2	2
21	Palpa	6	17	51	1	4	1	2
19	Palpa	8	25	30	3	6	7	0
22	Palpa	8	38	22	7	3	2	0
26	Palpa	8	19	33	9	11	0	1
12	DV	10	21	28	12	9	0	1
28	Palpa	16	18	32	9	1	2	2
11	Palpa	17	19	19	7	3	15	2
31	DV	19	8	1	7	1	43	1
42	DV	19	15	23	13	9	0	1
41	DV	20	30	16	11	2	0	0
25	Palpa	21	34	17	5	1	0	0
34	DV	23	10	30	13	4	0	0
10	DV	25	16	18	16	4	0	0
30	Palpa	25	26	17	6	4	0	0
29	Palpa	26	9	14	6	6	18	0
5	DV	33	30	6	6	4	0	0
9	DV	36	25	9	7	3	0	0
6	DV	37	14	4	21	0	3	0
14	Palpa	37	21	9	7	3	1	1
15	DV	39	15	9	16	1	0	0
8	DV	40	6	0	3	2	29	1
32	Palpa	40	21	8	6	3	1	1
43	DV	40	14	11	11	1	1	0
3	Palpa	42	19	6	10	2	0	0
33	Palpa	42	14	5	2	1	15	0
39	DV	49	17	1	11	1	0	1
24	Palpa	50	12	13	3	0	0	0
27	DV	60	8	0	11	1	0	0
1	DV	61	8	2	8	0	0	0
4	DV	62	11	0	6	1	0	0
2	DV	66	5	1	6	0	0	1
13	DV	67	7	0	4	1	1	2
35	DV	69	4	1	4	2	0	0
23	DV	70	7	0	2	1	0	0
36	DV	71	4	1	4	0	0	1
16	DV	72	4	0	3	0	1	1
37	DV	72	2	1	5	0	0	0
18	DV	73	3	1	3	0	0	1
40	DV	76	1	0	3	0	0	0
7	DV	78	2	0	0	0	0	2
38	DV	78	0	0	2	0	0	1
46	DV	80	0	0	0	0	0	0
Total (No.)		1805	637	651	304	112	143	28
			1288					

*Ordered by fewest correct responses*.

### Phonemic content of neologisms

**Figure 2:**
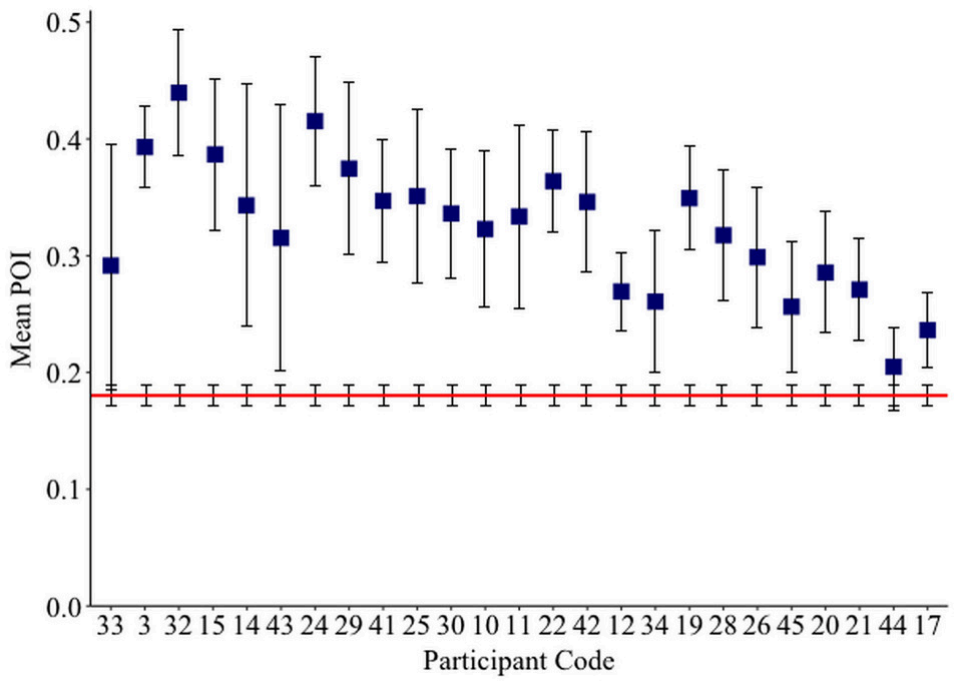
**Mean neologism Phonological Overlap Index (POI) score per Jargon individual (squares), and the mean chance POI estimate (red line)**. Error bars show 95% confidence intervals.

Chance POI was calculated as 0.18 (± 0.01) independent of the number of samples extracted from each null distribution (see Section Methods). The mean POI of neologisms produced by 23 Jargon individuals was greater than the chance prediction (*p* ≤ 0.007; see Table 3). Two individuals (33, 44) could not be differentiated from chance (*p* ≥ 0.066; see Figure 2).

**Table 3:** **Test statistics for Phonological Overlap Index (POI) and distribution analyses**.

**Pt code**	**Mean POI**	***p*****-value**	**KS stat^a^**
33	0.29	0.066	0.148
3	0.39	≤0.001	0.146
32	0.44	≤0.001	0.154
14	0.34	≤0.001	0.154
15	0.39	≤0.001	0.127
43	0.32	0.007	0.149
24	0.42	≤0.001	0.18
29	0.37	≤0.001	0.16
41	0.35	≤0.001	0.211^***^
25	0.34	≤0.001	0.109
30	0.35	≤0.001	0.163^**^
10	0.32	0.002	0.102
11	0.33	≤0.001	0.12
22	0.36	≤0.001	0.124^*^
42	0.35	≤0.001	0.121
12	0.27	0.003	0.212^***^
19	0.35	≤0.001	0.12
34	0.26	0.006	0.096
28	0.32	≤0.001	0.09
26	0.30	≤0.001	0.09
45	0.26	0.004	0.11
20	0.29	≤0.001	0.093
21	0.27	≤0.001	0.072
44	0.20	0.149	0.159^***^
17	0.24	≤0.001	0.09

* *p ≤ 0.05*;

** *p ≤ 0.01*;

*** *p ≤ 0.001*.

a *Kolmogorov-Smirnov test statistic*.

### Non-word accuracy distributions

The POI of all non-words (paraphasias and neologisms) produced by 20 neologistic individuals adhered to a normal distribution (*p* ≥ 0.067). Non-word POI distributions exhibited by individuals 41, 30, 22, 12, and 44, violated the normal distribution (0.124 ≤ KS ≤ 0.211; *p* ≤ 0.05; see Table 3). Individual 12 produced a bimodal distribution and individual 44 exhibited a left skew (see Supplementary Material). Histograms for these five individuals are presented in Supplementary Material.

### Perseveration results

**Figure 3:**
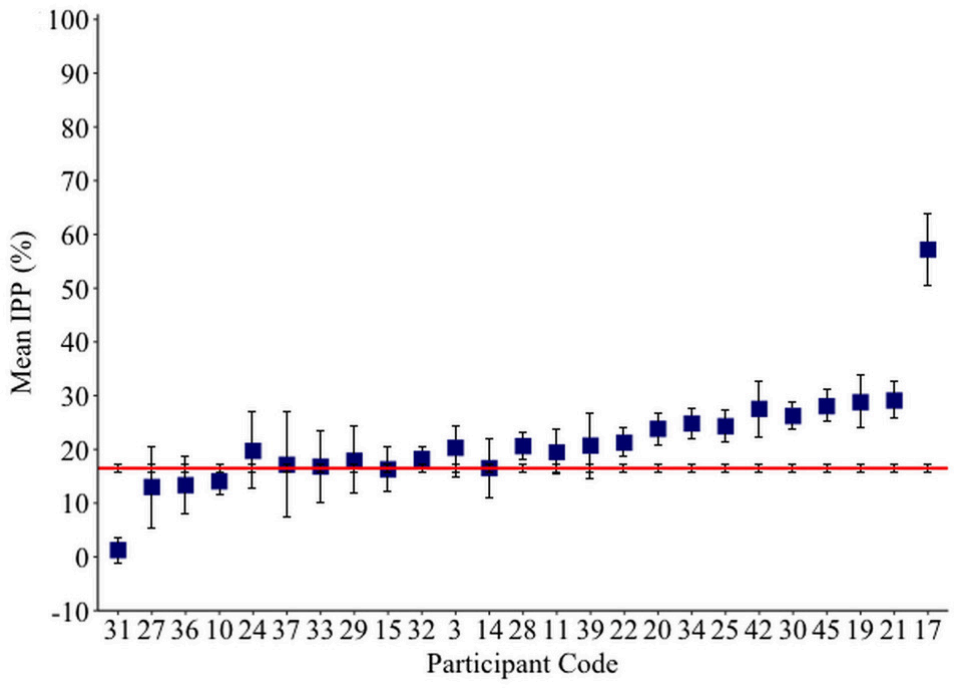
**Mean Intrusion Perseveration Probability (IPP) score per Jargon individual (square), and IPP chance estimate (red line)**. Error bars show 95% confidence intervals.

The IPP measure quantifies how frequently intruded phonemes occur over the previous 10 responses. This analysis was applied to individuals with fluent Jargon aphasia for whom suitable data were available (*n* = 25). The perseveration probability scores observed across lags 1–10 were averaged to derive a single IPP (perseveration) score. Individual IPP scores were compared against the null chance distributions. Thirteen individuals (3, 28, 39, 22, 20, 25, 34, 30, 42, 45, 19, 21, 17) produced perseveration at significantly greater rates than the chance prediction (*p* ≤ 0.039; see Table 4). The remaining 12 individuals did not perseverate at above the chance prediction (*p* ≥ 0.054; see Figure 3).

**Table 4:** **Test statistics for Intrusion Perseveration Probability (IPP) analysis**.

**Pt code**	**IPP mean**	***p*****-value**
31	0.01	1
27	0.13	0.963
36	0.13	0.949
10	0.14	0.905
15	0.16	0.527
14	0.17	0.467
33	0.17	0.395
37	0.17	0.337
29	0.18	0.269
32	0.18	0.219
11	0.19	0.068
24	0.20	0.054
3	0.20	0.039
28	0.21	0.029
39	0.21	0.028
22	0.21	0.007
20	0.24	≤ 0.001
25	0.24	≤ 0.001
34	0.25	≤ 0.001
30	0.26	≤ 0.001
42	0.28	≤ 0.001
45	0.28	≤ 0.001
19	0.29	≤ 0.001
21	0.29	≤ 0.001
17	0.57	≤ 0.001

### Relationship between perseverative and non-perseverative non-words

**Figure 4:**
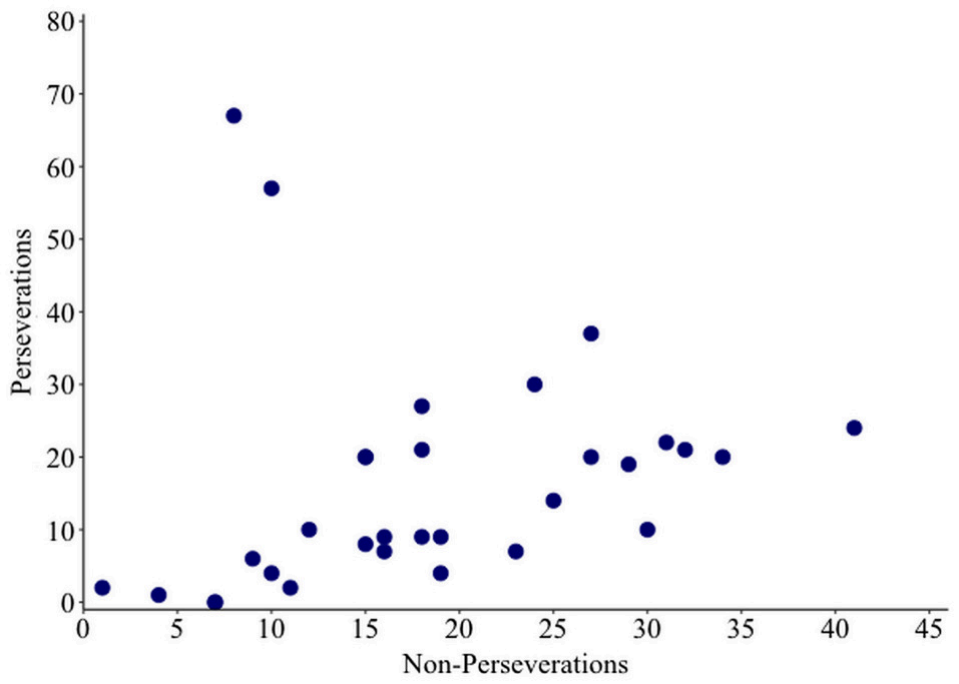
**Scatter plot showing the relationship between numbers of perseverative and non-perseverative non-words**.

Non-word errors were coded as a perseveration if an intruded phoneme was present in the previous response. Remaining non-words were coded as non-perseverative errors. A correlation analysis revealed a significant positive relationship between rates of perseverative and non-perseverative non-words (ρ = 0.557, *p* = 0.001; see Figure 4). The size of this effect increased from moderate to large when the two outlying individuals (17 and 44) were removed (ρ = 0.749, *p* ≤ 0.001).

### Lesion-symptom mapping

The Jargon aphasia group were combined with a wider aphasia group for whom neuroimaging data were available, to explore the relationship between lesion and jargon repetition. All but four participants (7; 38; 40; 46) in the wider aphasia group displayed a degree of repetition impairment; however, these impairments were only considered Jargon in 25 participants. As well as the significant relationship between perseverative and non-perseverative errors, Pearson correlation analyses displayed strong to medium relationships between overall repetition accuracy, number of neologistic errors, number of paraphasic errors and total number of intruded phonemes, see Table 5. Principal component analysis was used to derive a summary score representing number of neologisms, paraphasias and intruded phonemes (jargon score) which was entered into the VLSM analysis as the continuous dependent variable.

**Table 5:** **Correlations coefficients displaying medium-strong relationships between jargon score components**.

		**Number neologisms**	**Number paraphasias**	**Total intruded phonemes**
Repetition accuracy	*r-*value	−0.799	−0.709	−0.671
	*p-*value	<0.001	<0.001	<0.001
Number neologisms	*r-*value		0.363	0.851
	*p-*value		0.023	<0.001
Number paraphasias	*r-*value			0.324
	*p-*value			0.044

VLSM analysis identified lesion clusters associated with the jargon score in the posterior temporal and IPL, Figure 1C. These regions included the gray and white matter of the posterior STG, including areas Spt, the posterior superior temporal sulcus (STS), gray matter of the IPL including the SMG and white matter at the temporal-parietal boarder. These clusters remained significant at *p* = 0.01, (see Table 6); however they did not survive permutation correction.

**Table 6:** **Peak VLSM results, threshold ≤0.01**.

**Region**	**MNI coordinate**		
Posterior superior temporal gyrus	−42	−50	15
	−50	−36	17
	−58	−57	16
Superior temporal/inferior parietal lobe	−33	−37	15
Supramarginal gyrus	−50	−46	33
Posterior superior temporal sulcus	−45	−50	9

**Figure 5:**
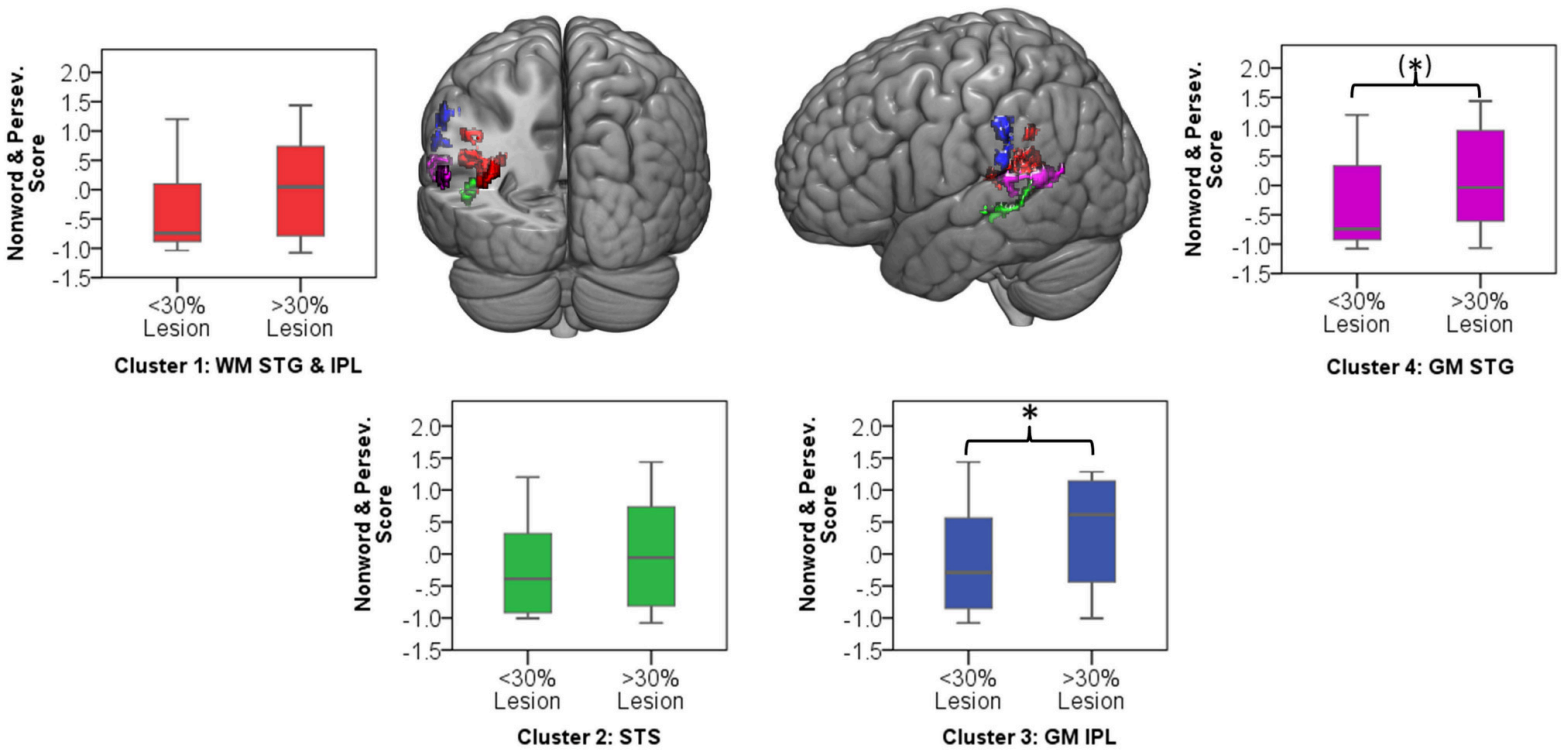
**Color areas display four regions of interest derived from VLSM clusters**. Graphs indicate jargon score for low and high lesion overlap group in each ROI. Ceiling performance on jargon score = −1.07. ^*^significant group difference; (^*^) borderline significant group difference. WM, white matter; GM, gray matter; STG, superior temporal gyrus; IPL, inferior parietal lobe; STS, superior temporal sulcus.

Significant clusters occurred in regions of high lesion overlap, (see Figure 1B). Therefore, follow-up ROI analyses were used to explore consistency of the VLSM results across the aphasia group. Four neuroanatomically constrained clusters were identified from the VLSM analyses: (1) White matter of the STG and IPL; (2) STS; (3) Gray matter of IPL including SMG and (4) Gray matter of the STG (see Figure 5). The number of lesion voxels in each ROI was identified for each participant and participants were separated into low overlap (<30% ROI voxels lesioned) or high overlap (>30% ROI voxels lesioned). *T*-tests were used to compare the jargon score between the high and low overlap groups in each ROI. There was no significant difference in jargon score for clusters 1 and 2. There was a significant difference in jargon scores between the high and low overlap groups in cluster 3, IPL [*t*_(36)_ = 2.0, *p* = 0.049], and a borderline significant difference in cluster 4, STG gray matter [*t*_(36)_ = 1.77, *p* = 0.085], (see Figure 5).

A regression analysis was performed to investigate whether a combination of lesions was most predictive of jargon production. The centered percentage lesion overlap of each ROI and the two-way interaction between ROIs were added as predictor variables alongside age, time post onset at testing and total lesion volume into a linear regression; jargon score was the dependent variable. Interaction terms were calculated by multiplying percentage of lesion in each cluster e.g., percentage overlap in cluster 1 × percentage overlap in cluster 2. Predictors in the model displayed sufficient collinearity tolerance; the minimum tolerance value outside interaction predictors was 0.2. The regression returned a borderline significant model [*F*_(12, 25)_ = 2.05, adjusted *R*^2^ = 0.253, *p* = 0.063]. Time post onset was a significant predictor (*t* = −2.3, *p* = 0.03) indicating that the greater time post onset the less jargon production. Lesions in isolated clusters did not significantly contribute to the model, however the interaction between cluster 2 (STS GM) and cluster 4 (STG GM) was a significant predictor (*t* = 2.3, *p* = 0.03) indicating that jargon was more severe when lesions affected both the STS and STG.

## Discussion

The aim of the current study was to explore, side by side, the behavioral and neurological patterns associated with repetition deficits in Jargon aphasia. Behavioral analyses identified the target relatedness of phonological distortions, and explored the effect of phoneme perseveration in Jargon repetition. Correlation analyses exposed the relationship between perseverative and non-perseverative errors. Lesion analyses were used to identify neurological regions and patterns of damage associated with jargon repetition. Results support the hypothesis that weak activation of target phonology results in neologistic production. Individuals with increasingly severe production deficits showed greater degrees of perseveration, and there was a clear association between the occurrence of perseverative and non-perseverative non-words, suggesting that both error types arise from a common mechanism. Lesion analyses converge with this interpretation and, additionally, implicate a contribution of impairments in analysis, and maintenance of auditory information to jargon repetition.

Psycholinguistic models account for non-word errors in Jargon aphasia through a breakdown in phonological encoding, whereby activation is not effectively transferred from the lexical to the phonological level (Schwartz et al., 2004; Marshall, 2006; Olson et al., 2007, 2015; Dell, 2014). Therefore, phonological and neologistic errors are accounted for by the same mechanism with differing degrees of breakdown severity. However, some evidence has pointed toward a random or default phonological activation pattern for some individuals, hypothesized to arise when lexical retrieval fails, (Butterworth, 1979; Moses et al., 2004; Eaton et al., 2010). Phonological Overlap Index (POI) analysis of the neologisms produced by the 25 participants with neologistic Jargon aphasia in the current study is largely consistent with the phonological encoding hypothesis and does not provide direct support for the default phonology hypothesis. The phonological overlap between neologisms and targets, although by definition low, was significantly above chance for 23 of the 25 neologistic Jargon aphasia participants, indicating a post-lexical retrieval breakdown. However, a large cluster of non-word errors with very limited target overlap occurring alongside errors with greater target relatedness would provide evidence for an additional lexical retrieval failure and default phonological production source. To investigate this hypothesis, the POI distribution across all non-word errors was analyzed. Only five individuals violated the normal distribution and only one of these participants (individual 12) displayed evidence of a separate cluster of non-word errors with limited target overlap, thus conforming to the two deficit account. However, caution must be taken in this interpretation in that the pattern could be accounted for by a large number of perseverative responses which were not distinguished within the POI or accuracy distribution analyses. For example, individual 44 exhibited a left skew indicating that most of their non-words had very limited target overlap and the POI analysis identified the accuracy of individual 44 as at chance. However, the correlation analysis indicated that individual 44 was highly perseverative, thus it is probable that their skewed POI distribution and neologistic accuracy is contaminated by perseveration.

A perseveration error is thought to occur when poor activation of target phonology allows recently used segments to compete and intrude. Therefore, perseveration errors are proposed to share a source with other non-word jargon errors (Martin and Dell, 2007; Buckingham and Buckingham, 2011). In the current study, 25 individuals with Jargon aphasia had suitable data for IPP perseveration analysis. Thirteen of these individuals displayed perseveration at a significantly greater level than the chance prediction, demonstrating that perseveration was a common but not universal feature of Jargon aphasia. Correlation analysis conformed to previous data (e.g., Martin and Dell, 2007) showing that non-word perseverative and non-perseverative error rates are strongly associated, indicating a common error source. Taken together, these results illustrate that perseverative errors occur at moderate to severe levels of phonological encoding impairment. One interpretation is that when phonological encoding is sufficiently impaired, a dearth of target activation results in the availability of only previously active phonological units. These results do not, however, preclude a breakdown of within-network inhibitory processes contributing to perseverative error production. Indeed, if the existence of both excitatory and inhibitory processes are presumed to occur within a cognitive system, it would be highly unlikely that one is impaired and the other spared.

Failure of inhibition as a dominant impairment is hypothesized to result in a qualitatively different error pattern than impairments in activating new target information, with consistent perseverative responses occurring without a correspondingly high level of non-perseverative non-word errors (Fischer-Baum and Rapp, 2012). Two individuals in the current study (participants 17 and 44) displayed this pattern, producing extremely high proportions of errors classified as perseverative with a comparatively low number of errors classified as non-perseverative non-word responses. This may indicate a greater contribution of inhibitory breakdown in these two individuals. Again, however, caution must be taken in this interpretation. The perseverative errors produced by these two individuals were blended perseverations in which responses contained both perseverated phonemes and non-perseverated phonemes. Non-perseverated phonemes were, for the most part, not related to the target item, suggestive of additional phonological encoding breakdown. Extreme breakdown in phonological encoding would cause consistent failure of target phonology activation and an over-reliance on previously encoded phonology, resulting in the majority of responses being identified as perseverative. This would also account for the error patterns produced by participants 17 and 44. Further testing of dissociating individuals would provide useful information on the nature and consistency of production patterns and is crucial for better understanding Jargon aphasia and the heterogeneity within the population (Nickels et al., 2011).

Voxel-lesion symptom mapping (VLSM) analyses were used to explore the relationship between lesion distribution and a sensitive measure of jargon repetition. Results parallel those obtained in previous VLSM studies and revealed a significant relationship between jargon production and lesion in the posterior temporoparietal region. Four significant clusters were identified in the gray matter of the pSTG, SMG, and superior temporal sulcus (pSTS) and the white matter at the border of the superior temporal and inferior parietal lobes. These regions are commonly observed to activate during functional imaging studies of speech production and repetition, although the precise roles remain under discussion. The pSTG region identified included area Spt at the border between the temporal and parietal lobe. Area Spt is proposed to be a hub region supporting the translation of auditory into motor information (Hickok and Poeppel, 2004; Warren et al., 2005; Buchsbaum et al., 2011; Hickok et al., 2011). These posterior auditory and phonological processes are thought to interact with frontal motor and articulatory processes via dorsal stream white matter tracts associated with the regions of white matter lesion identified in the current study. This finding converges with the phonological encoding impairment interpretation from the current and previous psycholinguistic analyses in that, in the context of repetition, phonological encoding requires the translation of auditory information into phonological patterns that can interface with articulatory processes. The SMG and pSTS regions identified in the VLSM analysis are associated with other processes. The SMG is frequently found to be active during tasks which require the temporary storage of phonological information, leading to the interpretation of this area as a phonological short term memory store. An impairment in phonological short term memory is likely to exacerbate difficulties with phonological encoding through a difficulty in maintaining phonological strings during production and, indeed, those with a greater degree of lesion in the SMG region displayed significantly more severe jargon repetition (Figure 5). The pSTS may play a role in maintaining auditory targets during repetition (Tourville et al., 2008; Markiewicz and Bohland, 2016). This converges with traditional hypotheses which implicate an impairment in self-monitoring in Jargon aphasia (Kinsbourne and Warrington, 1963; Maher et al., 1994); difficulties in holding auditory targets may result in limited information with which to monitor production. The VLSM analysis did not identify regions associated with articulatory processes, therefore indicating limited involvement of articulatory impairment in jargon repetition.

ROI analyses were used to explore whether combinations of lesions across the posterior temporal-parietal region were predictive of jargon repetition. Regression analysis found that combined lesions to the STG and STS region were associated with jargon production. This indicates that jargon is more likely to occur when impairments in phonological encoding and self-monitoring occur in combination. The STG and STS clusters were proximal and consequently there was a medium correlation between percentage lesion overlap in these clusters across the group (*r* = 0.54). However, over 1/3 of the group displayed high lesion overlap in the STG or STS but not in the other region, therefore this pattern is not fully accounted for by a lesion to a single region.

The VLSM analyses in the current study converge with previous lesion studies undertaken with a smaller proportion of severely impaired individuals. Therefore, these results indicate that jargon repetition may be a more severe manifestation of milder conduction-like repetition deficits. However, ROI analyses in the current study found that individuals with mild or no impairments still presented with lesions in regions identified by the VLSM analysis. These individual differences may be a consequence of post-stroke reorganization, which was also a significant predictor of jargon production and are of interest for neuroscientific studies of stroke recovery. These results should, however, be treated as exploratory. Although the results parallel previous VLSM studies of repetition in aphasia (Fridriksson et al., 2010; Baldo et al., 2012; Rogalsky et al., 2015), the results did not remain significant following permutation testing. This is likely to be a consequence of high lesion overlap in the aphasia group as a whole and the high prevalence of repetition impairment, Figure 1, Table 1. Additionally, caution must be taken in interpreting mass-univariate lesion-symptom mapping analyses which suffer from spatial distortion because of constraints of the vascular architecture (Mah et al., 2014) and do not account for regions which have limited functional capacity but remain structurally intact (Robson et al., in press).

### Insights for therapy

Current findings highlight several possible therapeutic strategies that may aid clinical management of Jargon aphasia. Weak activation of target segments at the phonological encoding level dictates that therapy and management should maximize the degree of activation feeding through to the phonological level. According to cognitive-neuropsychological models of word repetition, this is achieved via two converging avenues; lexical (via semantics) and sub-lexical (auditory-phonological analysis and translation into motor instructions). To fully utilize and maximize activation via lexical and sub lexical avenues, clinical tasks should include stimuli in multiple modalities, administering a written and verbal model of the stimuli and imagery where possible. Phonological awareness training could be adapted to include post phonological processing tasks—an area of comparative strength in this patient population (Romani et al., 2002; Romani and Galluzzi, 2005). Jargon aphasia therapy studies are scarce and further research is crucial to enhance understanding of the Jargon impairment and thus support development of targeted treatments.

## Conclusions

This study explored behavioral and neurological patterns associated with neologistic and perseverative word repetition errors in Jargon aphasia. Results from the behavioral and lesion analyses converge and support an impairment in encoding target phonology, possibly secondary to impairments in sensory-motor integration. Region of interest lesion analysis extended behavioral findings by indicating that impairments in maintaining auditory information in combination with phonological encoding impairments are particularly detrimental for repetition and were the most predictive of jargon responses in the current study. Behavioral analysis found that non-word and perseverative production are for the most part closely associated, paralleling previous psycholinguistic investigations and supporting the interpretation that perseverative and non-word errors can be accounted for by the same impairment source. These results imply that strengthening auditory-phonological integration and supporting self-monitoring would support speech production in Jargon aphasia.

## Ethics statement

Ethical approval for the current study was given by the Multicentre NHS Research Ethics Committee, the NHS East of England Research Ethics Committee and the University of Reading School of Psychology Research Ethics Committee. This study was carried out in accordance with the recommendations from the above Ethics committees, with written informed consent from all subjects. All subjects gave written informed consent in accordance with the Declaration of Helsinki.

## Author contributions

EP and HR: Research design, data collection, data analysis and interpretation, and manuscript preparation. JK: Research design, data analysis and interpretation, and manuscript preparation. LK: Data collection, data analysis and interpretation, and manuscript preparation. JS: Research design, data analysis support and manuscript preparation. KS: Research design, data collection, data interpretation, and manuscript preparation.

## Funding

This research was funded by a Stroke Association Postgraduate Fellowship awarded to EP (TSA PGF 2015-02), and a Stroke Association Senior Research Training Fellowship awarded to HR (TSA SRTF 2012/2).

### Conflict of interest statement

The authors declare that the research was conducted in the absence of any commercial or financial relationships that could be construed as a potential conflict of interest.

## References

[B1] Ackerman T. Ellis A. W. (2007). Case study: where do aphasic perseverations come from? Aphasiology 21, 1018–1038. 10.1080/02687030701198361

[B2] Anderson J. M. Gilmore R. Roper S. Crosson B. Bauer R. M. Nadeau S.. (1999). Conduction aphasia and the arcuate fasciculus: a reexamination of the Wernicke-Geschwind model. Brain Lang. 70, 1–12. 10.1006/brln.1999.2135 10534369

[B3] Baldo J. V. Katseff S. Dronkers N. F. (2012). Brain regions underlying repetition and auditory-verbal short-term memory deficits in aphasia: evidence from voxel-based lesion symptom mapping. Aphasiology 26, 338–354. 10.1080/02687038.2011.602391 24976669 PMC4070523

[B4] Basilakos A. Rorden C. Bonilha L. Moser D. Fridriksson J. (2015). Patterns of poststroke brain damage that predict speech production errors in apraxia of speech and aphasia dissociate. Stroke 46, 1561–1566. 10.1161/STROKEAHA.115.009211 25908457 PMC4442076

[B5] Bates E. Wilson S. M. Saygin A. P. Dick F. Sereno M. I. Knight R. T.. (2003). Voxel-based lesion-symptom mapping. Nat. Neurosci. 6, 448–450. 12704393 10.1038/nn1050

[B6] Boatman D. Gordon B. Hart J. Selnes O. Miglioretti D. Lenz F. (2000). Transcortical sensory aphasia: revisited and revised. Brain 123, 1634–1642. 10.1093/brain/123.8.1634 10908193

[B7] Bose A. (2013). Phonological therapy in Jargon aphasia: effects on naming and neologisms. Int. J. Lang. Commun. Disord. 48, 582–595. 10.1111/1460-6984.12038 24033655

[B8] Buchsbaum B. R. Baldo J. Okada K. Berman K. F. Dronkers N. D'Esposito M.. (2011). Conduction aphasia, sensory-motor integration, and phonological short-term memory – An aggregate analysis of lesion and fMRI data. Brain Lang. 119, 119–128. 10.1016/j.bandl.2010.12.001 21256582 PMC3090694

[B9] Buchsbaum B. R. Hickok G. Humphries C. (2001). Role of left posterior superior temporal gyrus in phonological processing for speech perception and production. Cogn. Sci. 25, 663–678. 10.1207/s15516709cog2505_2

[B10] Buckingham H. W. (1990). Abstruse neologisms, retrieval deficits and the random generator. J. Neurolinguistics 5, 215–235. 10.1016/0911-6044(90)90012-N

[B11] Buckingham H. W. Buckingham S. S. (2011). Is recurrent perseveration a product of deafferented functional systems with otherwise normal post-activation decay rates? Clin. Linguist. Phon. 25, 1066–1073. 10.3109/02699206.2011.616982 22106897

[B12] Buckingham H. W. Jr. Avakian-Whitaker H. Whitaker H. A. (1978). Alliteration and assonance in neologistic jargon aphasia. Cortex 14, 365–380. 10.1016/S0010-9452(78)80063-X 710147

[B13] Butterworth B. (1979). Hesitation and the production of verbal paraphasias and neologisms in Jargon aphasia. Brain Lang. 8, 133–161. 10.1016/0093-934X(79)90046-4 487066

[B14] Cohen L. Dehaene S. (1998). Competition between past and present. Assessment and interpretation of verbal perseverations. Brain 121, 1641–1659. 10.1093/brain/121.9.1641 9762954

[B15] Dell G. S. (2014). Phonemes and production. Lang. Cogn. Process. 29, 30–32. 10.1080/01690965.2013.851795 24443620 PMC3891789

[B16] Dell G. S. Martin N. Schwartz M. F. (2007). A case-series test of the interactive two-step model of lexical access: predicting word repetition from picture naming. J. Mem. Lang. 56, 490–520. 10.1016/j.jml.2006.05.007 21085621 PMC2981040

[B17] Dell G. S. Schwartz M. F. Martin N. Saffran E. M. Gagnon D. A. (1997). Lexical access in aphasic and nonaphasic speakers. Psychol. Rev. 104, 801–838. 10.1037/0033-295X.104.4.801 9337631

[B18] Deschamps I. Tremblay P. (2014). Sequencing at the syllabic and supra-syllabic levels during speech perception: an fMRI study. Front. Hum. Neurosci. 8:492. 10.3389/fnhum.2014.00492 25071521 PMC4086203

[B19] Eaton E. Marshall J. Pring T. (2010). Like deja vu all over again: patterns of perseveration in two people with Jargon aphasia. Aphasiology 24, 1017–1031. 10.1080/02687030903249343

[B20] Eaton E. Marshall J. Pring T. (2011). Mechanisms of change in the evolution of Jargon aphasia. Aphasiology 25, 1543–1561. 10.1080/02687038.2011.624584

[B21] Fischer-Baum S. Rapp B. (2012). Underlying cause(s) of letter perseveration errors. Neuropsychologia 50, 305–318. 10.1016/j.neuropsychologia.2011.12.001 22178232 PMC3259193

[B22] Fridriksson J. Kjartansson O. Morgan P. S. Hjaltason H. Magnusdottir S. Bonilha L.. (2010). Impaired speech repetition and left parietal lobe damage. J. Neurosci. 30, 11057–11061. 10.1523/JNEUROSCI.1120-10.2010 20720112 PMC2936270

[B23] Geschwind N. N. (1965). Disconnexion syndromes in animals and man. II. Brain 88:585. 5318824 10.1093/brain/88.3.585

[B24] Goodglass H. Kaplan E. Barresi B. (2001). The Boston Diagnostic Aphasia Examination: BDAE-3. Philadelphia, PA: Lippincott Williams & Wilkins.

[B25] Hanley J. R. Dell G. S. Kay J. Baron R. (2004). Evidence for the involvement of a nonlexical route in the repetition of familiar words: a comparison of single and dual route models of auditory repetition. Cogn. Neuropsychol. 21, 147–158. 10.1080/02643290342000339 21038197

[B26] Hanley J. R. Kay J. (1997). An effect of imageability on the production of phonological errors in auditory repetition. Cogn. Neuropsychol. 14, 1065–1084. 10.1080/026432997381277 20957537

[B27] Hanley J. R. Kay J. Edwards M. (2002). Imageability effects, phonological errors, and the relationship between auditory repetition and picture naming: implications for models of auditory repetition. Cogn. Neuropsychol. 19, 193–206. 10.1080/02643290143000132 20957537

[B28] Henson R. N. Burgess N. Frith C. D. (2000). Recoding, storage, rehearsal and grouping in verbal short-term memory: an fMRI study. Neuropsychologia 38, 426–440. 10.1016/S0028-3932(99)00098-6 10683393

[B29] Hickok G. (2009). The functional neuroanatomy of language. Phys. Life Rev. 6, 121–143. 10.1016/j.plrev.2009.06.001 20161054 PMC2747108

[B30] Hickok G. Buchsbaum B. Humphries C. Muftuler T. (2003). Auditory–motor interaction revealed by fMRI: speech, music, and working memory in area Spt. J. Cogn. Neurosci. 15, 673–682. 10.1162/089892903322307393 12965041

[B31] Hickok G. Houde J. Rong F. (2011). Sensorimotor integration in speech processing: computational basis and neural organization. Neuron 69, 407–422. 10.1016/j.neuron.2011.01.019 21315253 PMC3057382

[B32] Hickok G. Okada K. Serences J. T. (2009). Area Spt in the human planum temporale supports sensory-motor integration for speech processing. J. Neurophysiol. 101:2725. 10.1152/jn.91099.2008 19225172

[B33] Hickok G. Poeppel D. (2004). Dorsal and ventral streams: a framework for understanding aspects of the functional anatomy of language. Cognition 92, 67–99. 10.1016/j.cognition.2003.10.011 15037127

[B34] Hillis A. E. Caramazza A. (1991). Mechanisms for accessing lexical representations for output: evidence from a category-specific semantic deficit. Brain Lang. 40, 106–144. 10.1016/0093-934X(91)90119-L 2009445

[B35] Hirsh K. W. (1998). Perseveration and activation in aphasic speech production. Cogn. Neuropsychol. 15, 377–388. 10.1080/026432998381140 28657506

[B36] Hodges J. R. Martinos M. Woollams A. M. Patterson K. Adlam A.-L. (2008). Repeat and point: differentiating semantic dementia from progressive non-fluent aphasia. Cortex 44, 1265–1270. 10.1016/j.cortex.2007.08.018 18761140

[B37] Isenberg A. L. Vaden K. I. Saberi K. Muftuler L. T. Hickok G. (2012). Functionally distinct regions for spatial processing and sensory motor integration in the planum temporale. Hum. Brain Mapp. 33, 2453–2463. 10.1002/hbm.21373 21932266 PMC5242090

[B38] Itabashi R. Nishio Y. Kataoka Y. Yazawa Y. Furui E. Matsudaand M.. (2016). Damage to the left precentral gyrus is associated with apraxia of speech in acute Stroke 47, 31–36. 10.1161/STROKEAHA.115.010402 26645260

[B39] Jefferies E. Lambon Ralph M. A. (2006). Semantic impairment in stroke aphasia versus semantic dementia: a case-series comparison. Brain 129, 2132–2147. 10.1093/brain/awl153 16815878

[B40] Kaplan E. (1983). The Assessment of Aphasia and Related Disorders, Vol. 2 Philadelphia, PA: Lippincott Williams & Wilkins.

[B41] Kay J. Lesser R. Coltheart M. (1996). Psycholinguistic assessments of language processing in aphasia (PALPA): an introduction. Aphasiology 10, 159–180. 10.1080/02687039608248403

[B42] Kinsbourne M. Warrington E. K. (1963). Jargon aphasia. Neuropsychologia 1, 27–37. 10.1016/0028-3932(63)90010-1

[B43] Mah Y.-H. Husain M. Rees G. Nachev P. (2014). Human brain lesion-deficit inference remapped. Brain 137, 2522–2531. 10.1093/brain/awu164 24974384 PMC4132645

[B44] Maher L. M. Rothi L. J. G. Heilman K. M. (1994). Lack of error awareness in an aphasic patient with relatively preserved auditory comprehension. Brain Lang. 46, 402–418. 10.1006/brln.1994.1022 7514943

[B45] Markiewicz C. J. Bohland J. W. (2016). Mapping the cortical representation of speech sounds in a syllable repetition task. Neuroimage 141, 174–190. 10.1016/j.neuroimage.2016.07.023 27421186

[B46] Marshall J. (2006). Jargon aphasia: what have we learned? Aphasiology 20, 387–410. 10.1080/02687030500489946

[B47] Marshall J. Robson J. Pring T. Chiat S. (1998). Why does monitoring fail in Jargon aphasia? Comprehension, judgment, and therapy evidence. Brain Lang. 63, 79–107. 10.1006/brln.1997.1936 9642022

[B48] Martin N. (1996). Models of deep dysphasia. Neurocase 2, 73–80. 10.1080/13554799608402391

[B49] Martin N. Dell G. S. (2007). Common mechanisms underlying perseverative and non-perseverative sound and word substitutions. Aphasiology 21, 1002–1017. 10.1080/02687030701198346

[B50] Martin N. Dell G. S. Saffran E. M. Schwartz M. F. (1994). Origins of paraphasias in deep dysphasia: testing the consequences of a decay impairment to an interactive spreading activation model of lexical retrieval. Brain Lang. 47, 609–660. 10.1006/brln.1994.1061 7859057

[B51] McCarthy R. Warrington E. K. (1984). A two-route model of speech production. Brain 107:463. 10.1093/brain/107.2.463 6722512

[B52] McGettigan C. Warren J. E. Eisner F. Marshall C. R. Shanmugalingam P. Scott S. K. (2010). Neural correlates of sublexical processing in phonological working memory. J. Cogn. Neurosci. 23, 961–977. 10.1162/jocn.2010.21491 20350182 PMC3376447

[B53] Mesgarani N. Cheung C. Johnson K. Chang E. (2014). Phonetic feature encoding in human superior temporal gyrus. Science 343, 1006–1010. 10.1126/science.1245994 24482117 PMC4350233

[B54] Moritz-Gasser S. Duffau H. (2013). The anatomo-functional connectivity of word repetition: insights provided by awake brain tumor surgery. Front. Hum. Neurosci. 7:405. 10.3389/fnhum.2013.00405 23908617 PMC3725408

[B55] Moses M. S. Nickels L. A. Sheard C. (2004). Disentangling the web: neologistic perseverative errors in Jargon aphasia. Neurocase 10, 452–461. 10.1080/13554790490894057 15788285

[B56] Moses M. S. Nickels L. A. Sheard C. (2007a). Chips, cheeks and carols: a review of recurrent perseveration in speech production. Aphasiology 21, 960–974. 10.1080/02687030701198254

[B57] Moses M. S. Sheard C. Nickels L. A. (2007b). Insights into recurrent perseverative errors in aphasia: a case series approach. Aphasiology 21, 975–1001. 10.1080/02687030701198312

[B58] Nickels L. Howard D. Best W. (2011). On the use of different methodologies in cognitive neuropsychology: drink deep and from several sources. Cogn. Neuropsychol. 28, 475–485. 10.1080/02643294.2012.672406 22746689 PMC3996528

[B59] Nozari N. Kittredge A. K. Dell G. S. Schwartz M. F. (2010). Naming and repetition in aphasia: steps, routes, and frequency effects. J. Mem. Lang. 63, 541–559. 10.1016/j.jml.2010.08.001 21076661 PMC2976549

[B60] Olson A. C. Romani C. Halloran L. (2007). Localizing the deficit in a case of jargon aphasia. Cogn. Neuropsychol. 24, 211–238. 10.1080/02643290601137017 18416489

[B61] Olson A. Halloran E. Romani C. (2015). Target/error overlap in jargon aphasia: the case for a one-source model, lexical and non-lexical summation, and the special status of correct responses. Cortex 73, 158–179. 10.1016/j.cortex.2015.06.028 26410740

[B62] Panzeri M. Semenza C. Butterworth B. (1987). Compensatory processes in the evolution of severe Jargon aphasia. Neuropsychologia 25, 919–933. 10.1016/0028-3932(87)90096-0

[B63] Papagno C. Basso A. (1996). Perseveration in Two aphasic patients. Cortex 32, 67–82. 10.1016/S0010-9452(96)80017-7 8697753

[B64] Paulesu E. Frith C. D. Frackowiak R. S. (1993). The neural correlates of the verbal component of working memory. Nature 362, 342–345. 10.1038/362342a0 8455719

[B65] Quigg M. Fountain N. B. (1999). Conduction aphasia elicited by stimulation of the left posterior superior temporal gyrus. J. Neurol. Neurosurg. Psychiatr. 66, 393–396. 10.1136/jnnp.66.3.393 10084542 PMC1736266

[B66] Ravizza S. M. Delgado M. R. Chein J. M. Becker J. T. Fiez J. A. (2004). Functional dissociations within the inferior parietal cortex in verbal working memory. Neuroimage 22, 562–573. 10.1016/j.neuroimage.2004.01.039 15193584

[B67] Robson H. Cloutman L. Keidel J. L. Sage K. Drakesmith M. Welbourne S. (2014). Mismatch negativity (MMN) reveals inefficient auditory ventral stream function in chronic auditory comprehension impairments. Cortex 59, 113–125. 10.1016/j.cortex.2014.07.009 25173955

[B68] Robson H. Grube M. Lambon Ralph M. A. Griffiths T. D. Sage K. (2013). Fundamental deficits of auditory perception in Wernicke's aphasia. Cortex 49, 1808–1822. 10.1016/j.cortex.2012.11.012 23351849

[B69] Robson H. Keidel J. L. Ralph M. A. Sage K. (2012). Revealing and quantifying the impaired phonological analysis underpinning impaired comprehension in Wernicke's aphasia. Neuropsychologia 50, 276–288. 10.1016/j.neuropsychologia.2011.11.022 22172546

[B70] Robson H. Specht K. Beaumont H. Parkes L. M. Sage K. Lambon Ralph M. A. (in press). Arterial spin labelling shows functional depression of non-lesion tissue in chronic Wernicke's aphasia. Cortex. 10.1016/j.cortex.2016.11.002. PMC5480775 28525836

[B71] Robson J. O. Marshall J. Pring T. I. M. Chiat S. (1998a). Phonological naming therapy in Jargon aphasia: positive but paradoxical effects. J. Int. Neuropsychol. Soc. 4, 675–686. 10.1017/S1355617798466153 10050371

[B72] Robson J. O. Pring T. Marhsall J. Morrison S. Chiat S. (1998b). Written communication in undifferentiated Jargon aphasia: a therapy study. Int. J. Lang. Commun. Disord. 33:305. 10.1080/136828298247767 10326042

[B73] Rogalsky C. Poppa T. Chen K.-H. Anderson S. W. Damasio H. Love T.. (2015). Speech repetition as a window on the neurobiology of auditory–motor integration for speech: a voxel-based lesion symptom mapping study. Neuropsychologia 71, 18–27. 10.1016/j.neuropsychologia.2015.03.012 25777496 PMC4417364

[B74] Romani C. Galluzzi C. (2005). Effects of syllabic complexity in predicting accuracy of repetition and direction of errors in patients with articulatory and phonological difficulties. Cogn. Neuropsychol. 22, 817–850. 10.1080/02643290442000365 21038278

[B75] Romani C. Olson A. Semenza C. Grana A. (2002). Patterns of phonological errors as a function of a phonological versus an articulatory locus of impairment. Cortex 38, 541–567. 10.1016/S0010-9452(08)70022-4 12465668

[B76] Rorden C. Bonilha L. Fridriksson J. Bender B. Karnath H.-O. (2012). Age-specific CT and MRI templates for spatial normalization. Neuroimage 61, 957–965. 10.1016/j.neuroimage.2012.03.020 22440645 PMC3376197

[B77] Sandson J. Albert M. L. (1984). Varieties of perseveration. Neuropsychologia 22, 715–732. 10.1016/0028-3932(84)90098-8 6084826

[B78] Santo Pietro M. J. Rigrodsky S. (1986). Patterns of oral-verbal perseveration in adult aphasics. Brain Lang. 29, 1–17. 10.1016/0093-934X(86)90030-1 3756452

[B79] Schwartz M. F. Wilshire C. E. Gagnon D. A. Polansky M. (2004). Origins of nonword phonological errors in aphasic naming. Cogn. Neuropsychol. 21, 159–186. 10.1080/02643290342000519 21038198

[B80] Stark J. (2007). A review of classical accounts of verbal perseveration and their modern-day relevance. Aphasiology 21, 928–959. 10.1080/02687030701198239

[B81] Tourville J. A. Reilly K. J. Guenther F. H. (2008). Neural mechanisms underlying auditory feedback control of speech. Neuroimage 39, 1429–1443. 10.1016/j.neuroimage.2007.09.054 18035557 PMC3658624

[B82] Trébuchon A. Démonet J. Chauvel P. Liégeois-Chauvel C. (2013). Ventral and dorsal pathways of speech perception: an intracerebral ERP study. Brain 127, 273–283. 10.1016/j.bandl.2013.04.007 24028995

[B83] Warren J. E. Wise R. J. Warren J. D. (2005). Sounds do-able: auditory–motor transformations and the posterior temporal plane. Trends Neurosci. 28, 636–643. 10.1016/j.tins.2005.09.010 16216346

[B84] Yamadori A. (1981). Verbal perseveration in aphasia. Neuropsychologia 19, 591–594. 10.1016/0028-3932(81)90026-9 6168969

