# Supplementary material for: Sources of Phoneme Errors in Repetition: Perseverative, Neologistic, and Lesion Patterns in Jargon Aphasia
Source: Front Hum Neurosci. 2017 May 4;11:225. doi: 10.3389/fnhum.2017.00225 (PMC5415595; doi:10.3389/fnhum.2017.00225)
Supplement: Supplementary file 1 [file DataSheet1.DOCX]

***Supplementary Material***

**Sources of Phoneme Errors in Repetition: Perseverative, Neologistic and Lesion Patterns in Jargon aphasia**

Emma Pilkington^1^, James Keidel^2^, Luke Thomas Kendrick^1^, James Douglas Saddy^1^, Karen Sage^3*^, Holly Robson^1^

*** Correspondence:** Professor Karen Sage: [k.sage@shu.ac.uk](mailto:k.sage@shu.ac.uk)


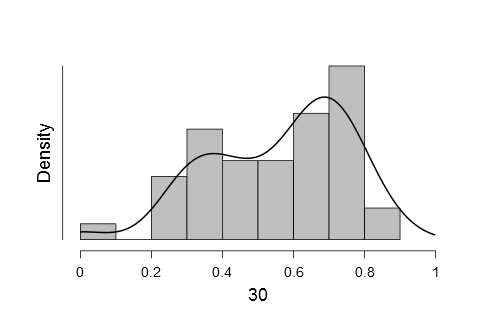
 A B


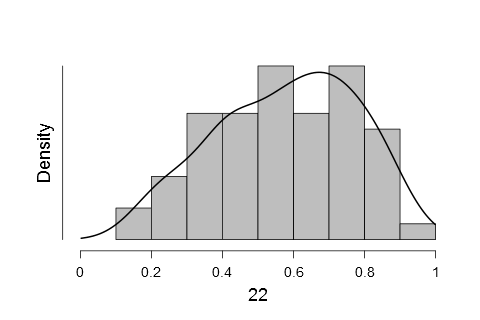


C D


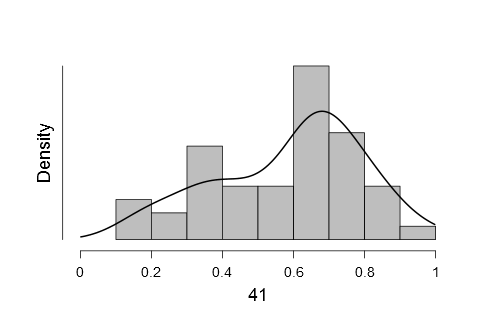

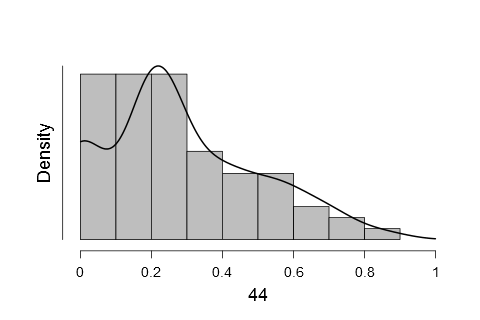


E


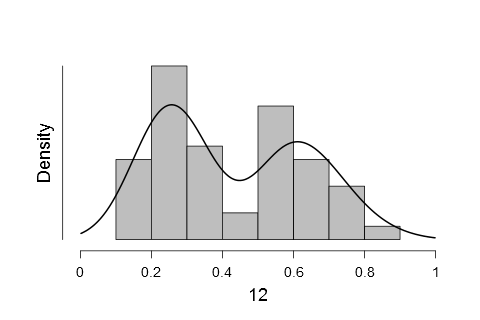


Phonological Overlap Index (POI) histograms for non-words produced by five neologistic Jargon individuals. **(A)** individual 30, **(B)** individual 22; **(C)** individual 41; **(D)** individual 44; **(E)** individual 12.
